# Rebamipide ameliorates atherosclerosis by controlling lipid metabolism and inflammation

**DOI:** 10.1371/journal.pone.0171674

**Published:** 2017-02-27

**Authors:** JooYeon Jhun, Jeong-Eun Kwon, Se-Young Kim, Jeong-Hee Jeong, Hyun Sik Na, Eun-Kyung Kim, Seung Hoon Lee, KyungAh Jung, Jun-Ki Min, Mi-La Cho

**Affiliations:** 1 The Rheumatism Research Center, Catholic Research Institute of Medical Science, The Catholic University of Korea, Seoul, South Korea; 2 IMPACT Biotech, Catholic Research Institute of Medical Science, The Catholic University of Korea, Seoul, South Korea; 3 Bucheon St. Mary’s Hospital, Division of Rheumatology, Department of Internal Medicine, College of Medicine, The Catholic University of Korea, Bucheon, South Korea; Seoul National University College of Pharmacy, REPUBLIC OF KOREA

## Abstract

Atherosclerosis is a chronic inflammatory disease caused by the accumulation of excess lipid in the aorta and the severity is regulated by T lymphocytes subsets. Rebamipide has therapeutic activity in collagen induced arthritis (CIA) by controlling the balance between T helper (Th) 17 and regulatory T (Treg) cells. In this study, we aimed to determine whether rebamipide reduces the development of atherosclerosis. To investigate the therapeutic effect of rebamipide, ApoE-KO mice fed a western diet were administered rebamipide orally for 8 weeks. Mice were sacrificed followed by the evaluation of plaque formation in the aorta or immunohistochemistry for IL-17 and Foxp3. Serum was also prepared to determine the pro-inflammatory cytokine levels. The ability of rebamipide to regulate lipid metabolism or inflammation was confirmed *ex vivo*.

**Results:**

The oral administration of rebamipide decreased plaque formation in atherosclerotic lesions as well as the markers of metabolic disorder in ApoE-deficient mice with atherosclerosis. Pro-inflammatory cytokines were also suppressed by rebamapide. In addition, the population of Th17 was decreased, whereas Treg was increased in the spleen of rebamipide-treated ApoE deficient mice. Rebamipide also ameliorated the severity of obese arthritis and has the capability to reduce the development of atherosclerosis by controlling the balance between Th17 and Treg cells. Thus, rebamipide could be a therapeutic agent to improve the progression of inflammation in metabolic diseases.

## Introduction

Atherosclerosis is an inflammatory arterial disorder mediated by chronic immune responses and related by activated T cells. Atherosclerosis reportedly involves the thickening of the artery wall as a result of the infiltration and accumulation of CD4^+^ T cells, which are involved in plaque formation [1, 2]. In fact, CD4^+^ T cells increase the progression of atherosclerosis in Apolipoprotein E (ApoE) knockout mice [3].

The initial stimulation of the vascular tissue recruits and activates monocytes to differentiate into macrophage or dendritic cells, followed by the recruitment of helper T (Th) cells such as Th1, Th2 or Th17 cells [4–6]. Th17, an inflammatory mediator in many autoimmune diseases [7–9], was detected in the aortic sinus of *Ldlr*^-/-^mice and was found to be increased in ApoE knockout mice on a western diet [6, 10]. In addition, upregulation of Th17 cells increased atherosclerotic plaque in experimental atherosclerosis [11]. IL-17 induces vascular and systemic inflammatory responses, and blockade of IL-17A reduces the formation of atherosclerotic lesions in ApoE knockout mice [12, 13]. Reciprocal regulation by Th17 and regulatory T (Treg) cells has a key role in atherogenesis in ApoE knockout mice [14].

Rebamipide, an amino acid derivative of 2-(1H)-quinolinone, is used as a gastroprotective drug for treating ulcers and shows anti-inflammatory activity in gastric ulcers [15]. Recently, rebamipide was shown to improve dermatitis by decreasing inflammation in skin and was also found to suppress the development of autoimmune arthritis by downregulating inflammation [16, 17]. Rebamipide controls the balance between Th17 and Treg cells in experimental arthritis [18].

We hypothesized that rebamipide suppresses atherosclerosis development through reciprocal regulation of Th17/Treg. To identify the therapeutic activity of rebamipide, we induced atherosclerosis in ApoE knockout mice and determined the atherogenesis reducing activity of rebamipide by evaluating the levels of inflammatory cytokines or serum lipids *in vivo* or *in vitro*.

## Material and methods

### Mice and diet

Seven week old male apolipoprotein E (ApoE)-knockout mice (Jackson Laboratory, Bar Harbor, ME, USA) were fed a western diet containing 45% calories from fat and 1.5% cholesterol (Ralston Purina, St Louis, MO). Four-week-old male c57BL/6 mice (Orient Bio, Republic of Korea) were housed in polycarbonate cages. They were fed 60Kcal fat-derived calories and standard mouse chow (Ralston Purina, St Louis, MO) with water provided ad *libitum*. All the experimental procedures were approved by the animal Ethics Committee of the Catholic University of Korea.

### Induction of CIA

CII immunization was initially performed when the mouse weighed 30gram. In brief, an emulsion was formed by dissolving 2mg/ml chick collagen type II (CII; Chondrex, Redmone, WA) overnight at 4°C in 0.5M acetic acid, followed by mixing with an equal volume of CFA (Chondrex, Redmond, WA). The mice were intradermally injected with the emulsion at 2 sites: at the base of the tail and a at slightly more anterior location. A second injection was administered as a booster 14 days after the primary immunization.

### Clinical assessment of arthritis

The severity of arthritis was determined by 3 independent observers. The mice were evaluated once a week for the onset and severity of joint inflammation for up to 15 weeks after the primary immunization. The severity of arthritis was assessed on a scale of 0–4 with the following criteria, as described previously: 0 = no edema or swelling, 1 = slight edema and erythema limited to the foot or ankle, 2 = slight edema and erythema from the ankle to the tarsal bone, 3 = moderate edema and erythema from the ankle to the tarsal bone and 4 = edema and erythema from the ankle to the entire leg. The arthritis score for each mouse was expressed as the sum of the scores for 3 limbs.

### Histological analysis of arthritis

The mouse joint tissues were fixed with 10% formalin, decalcified in EDTA and embedded in paraffin. The sections were deparaffinized using xylene and dehydrated in a graded series of alcohol solutions. The sections were then stained with Harris hematoxylin and Eosin (H&E), Safranin O and Toluidine blue to detect proteoglycans. The H&E stained sections were scored for inflammation according to the following criteria: 0 = no inflammation, 1 = slight thickening of the lining layer or some infiltrating cells in the underlying layer, 2 = slight thickening of the lining layer plus some infiltrating cells in the underlying layer, 3 = thickening of the lining layer, an influx of cells in the underlying layer and the presence of cells in the synovial space and 4 = synovium infiltrated by many inflammatory cells. Cartilage damage was determined using safranin-O staining and Toluidine blue and the extent of cartilage damage was scored according to the following criteria: 0 = no destruction, 1 = minimal erosion limited to single spots, 2 = slight to moderate erosion in a limited area, 3 = more extensive erosion and 4 = general destruction.

### Aortic atherosclerotic analysis

Whole aortas were excised proximal to the aortic arch and femoral bifurcation. Aortas were fixed with 10% formalin overnight and stored in PBS at 4°C. Aortas were cleaned of extraneous fat and tissue, longitudinally dissected and stained with 60% Oil red O. The stained aorta tissues were kept in fixation solution until images were captured. Images were captured digitally using a ColorView 12 CCD attached to an Olympus BX-51 with a 1.25x objective lens and a wide-angle condenser.

All of the aortic histologic images corresponding to the shear wave elastographic region of interests were transformed into digital microscopic images by a scanning system at high resolutions and converted into MRXS files by slide Converter (3DHISTECH, Budapest, Hungary). Computer graphic analysis was performed with Pannoramic Viewer and HistoQuant software (3DHISTECH) under detailed measurement settings Note that the analyzed area on shear wave elastography was the same size as that on histologic examination. The upper half of the heart with the ascending aorta was dissected and paraffin-embedded. Sequential 5 *μ*m thick sections were cut from the heat toward the aortic root and stained with H&E.

### Foam cell formation assay

An in vitro foam cell formation assay was performed using THP-1 cells. The THP-1 human monocyte derived cell line was purchased from the American Type Culture Collection (ATCC, Manassas, VA, USA), and cultured in RPMI-1640 medium containing 10% fetal bovine serum (FBS). Differentiation into macrophages was achieved by treating the cells with 160nM phorbol 12-myristate 13-acetate (PMA) for 48hours at 37°C. The aorta were stained with 60% Oil red O.

### Immunohistochemistry analysis of brachiocephalic plaques

Brachiocephalic arteries were post-fixed in 10% formalin, embedded in paraffin and sectioned at 5μm intervals. Sections adjacent to the point of maximum vessel occlusion were used for the comparison of plaque composition. Immunohistochemistry was performed using the Vectastain ABC kit (Vector Laboratories, Burlingame, CA, USA). The tissue was first incubated with primary antibodies to IL-17, Foxp3 and VCAM-1 (Santa Cruz Biotechnology, Santa Cruz, CA, USA) overnight at 4°C. The sections were counterstained with hematoxylin. Samples were photographed with an Olympus photomicroscope (Tokyo, Japan).

### Biochemical parameters

Blood samples from treated and control mice were collected after the consumption of a western diet for 8 weeks and stored at -70°C until use. The levels of total serum cholesterol and LDL-cholesterol were measured using commercial kits from Wako Co. (Osaka, Japan), and triglyceride, aspartate aminotransferase (AST) and alanine aminotransferase (ALT) were measured using commercial kits from Asan Pharmaceutical Co. (Hwaseong-si Gyeonggi-do, Korea)

### Flow cytometry

Cells were stained with Percp-conjugated anti-CD4 Ab (BD Pharmingen), then stained with APC-conjugated anti-CD25, PE conjugated anti-Foxp3 and FITC-conjugated anti IL-17 (all from eBiosciences, San Diego, CA, USA), followed by fixation and permeabilization using the Buffer Set (BD Biosciences) according to the manufacturer's instructions. All samples were run on a FACSCalibur (BD Pharmingen), and data were analyzed using the FlowJo software (Tree Star, Ashland, OR, USA).

### Confocal microscopy

For confocal staining, 7 mM tissue sections of spleens were stained using PE-conjugated anti-CD4, FITC conjugated anti-IL-17, Foxp3 conjugated anti-FITC and APC conjugated anti-CD25 (all from eBiosciences). Stained sections were analyzed using a Zeiss microscope (LSM 510 Meta; Carl Zeiss, Oberkochen, Germany) at x400 magnification.

### Splenocyte culture and stimulation

The spleens were isolated from the DBA1/J mice. The spleens were minced and the red blood cells were lysed with 0.83% ammonium chloride. The cells were filtered through a cell strainer and centrifuged at 1300 rpm at 4°C for 5 min. A single suspension was prepared, and 5x10^5^ cells/well in 48-well flat bottom plates were cultured in the presence of 100ng/ml lipopolysaccharide (LPS), 20μM Rebamipide, and 100μM Rebamipide for 2 days.

### Statistical analysis

All data were expressed as the mean±standard deviation (SD). Experimental values were presented as mean±SD of triplicate cultures and 3 representative experiments. Statistical significance was determined by the Mann–Whitney U-test or ANOVA with

Bonferroni’s post hoc test using GraphPad Prism (version 5.01, GraphPad Software, San Diego, CA). Values of p0.05 were considered statistically significant.

## Results

### Rebamipide ameliorates atherosclerosis and recovers lipid metabolism in ApoE-KO mice

**Fig 1:**
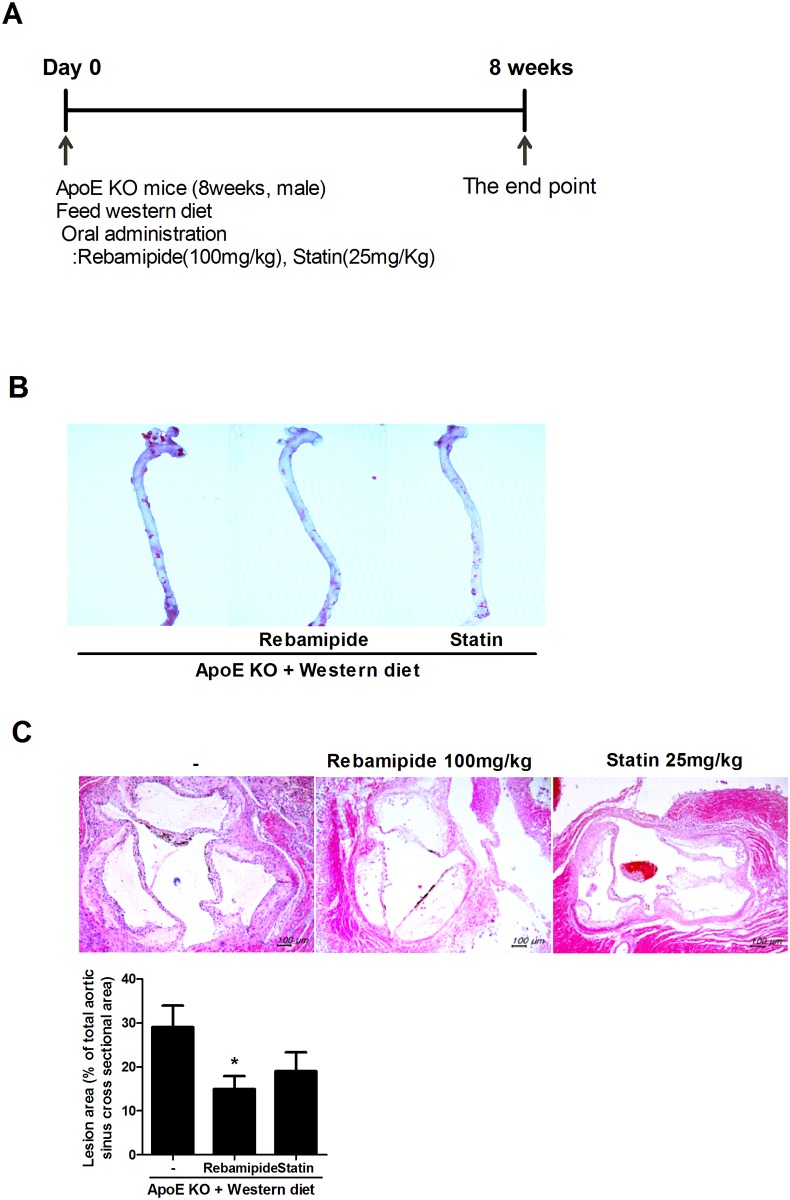
Rebamipide decreased atherosclerotic lesions in ApoE-KO mice. (A) To induce atherosclerosis, eight-week old male ApoE-KO mice were fed a western diet with rebamipide (100 mg/kg/day) or statin (25mg/kg/day) every day for 8 weeks. (B) Aorta were prepared from the ApoE-KO mice at the end of study and stained with Oil-red-O to determine the accumulation of lipid. (C) Longitudinal section of aorta from rebamipide or statin administrated mouse was stained with Oil red O and lesion area was analyzed.

To investigate the effects of rebamipide on atherosclerosis, we fed a western diet to ApoE–deficient (ApoE-KO) mice to induce atherosclerosis and administered rebamipide or statin, used as the positive control (Fig 1A). Rebamipide and statin ameliorated plaque formation in the aortas of ApoE-KO mice on a western diet as compared to the negative control (Fig 1B). To quantify the effect of rebamipide or statin, we performed the longitudinal dissection of aorta from each group. Fig 1C showed lesion area of aorta and graph means that both of rebamipide and statin decreased the lesion area, but rebamipide showed greater effect than statin.

**Fig 2:**
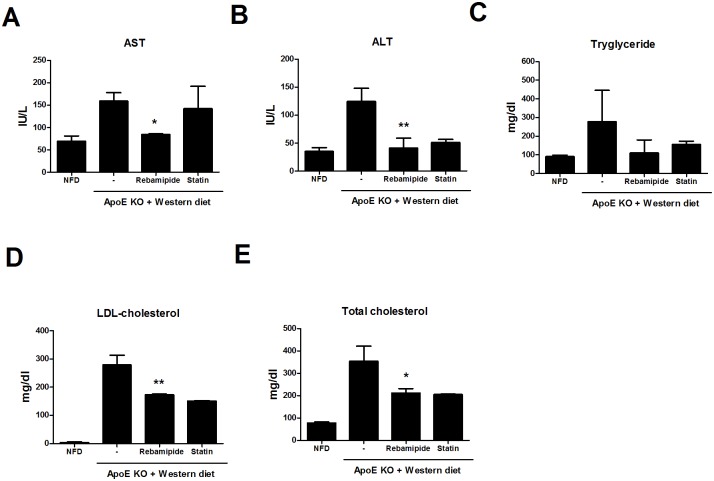
Rebamipide therapy decreased serum lipid in the ApoE KO. (A)-(E) Metabolic parameters from atherosclerosis-induced ApoE-KO mice that were orally administrated with or without rebamipide or statin were measured using blood at the 8^th^ week. Aspartate aminotransferase (AST), Alanine aminotrasferase (ALT). NFD (non-fat diet).

**Fig 3:**
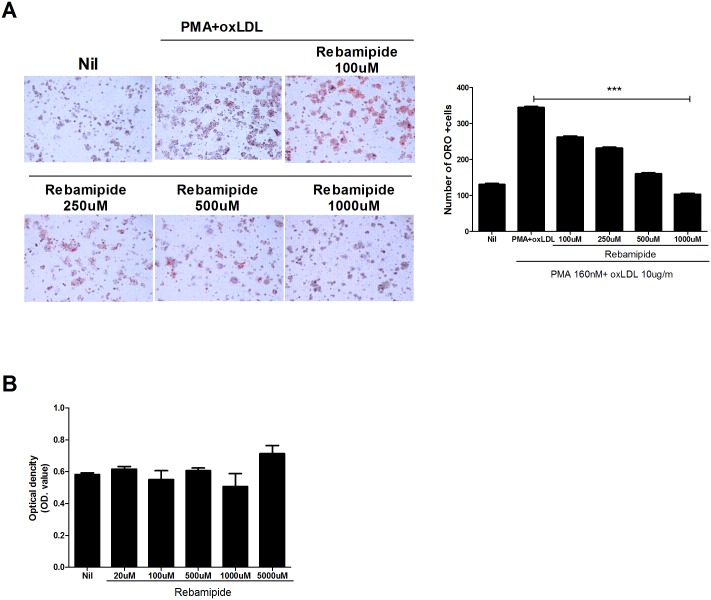
Inhibitory effect of rebamipide on ox-LDL induced foam cell formation. (A) THP-1 cells were incubated with PMA for 1 day and then with oxLDL followed by co-treatment with rebamipide at a dose of 100, 250, 500 and 1000 μM as indicated. Twenty-four hours later, the accumulation of lipid droplets was observed using oil red O staining. The graph shows the number of oil red O stained cells counted. (B) The cell viability of THP-1 cells was also determine through MTT assay. Graph shows O.D. for each indicated rebamipide concentration.

To confirm the lipid-control function of rebamapide, liver cell injury was also tested. The administration of rebamipide showed decreased levels of aspartate transaminase (AST) and transaminase (ALT), markers of liver damage and hepatotoxicity due to high lipid levels (Fig 2A and 2B). As the severity of atherosclerosis is related with the amount of inflammation or lipids in the serum, we first determined the levels of bio-metabolic factors such as triglyceride (TAG), low-density lipoprotein (LDL)-cholesterol and total cholesterol in the blood sera of ApoE-KO mice. Decreased levels of TAG and cholesterol by rebamipide or statin were suggestive of potential anti-hyperlipidemia function (Fig 2C–2E). These data indicated that rebamapide improved lipid-metabolic factors in the atherosclerosis mouse model. Further investigation was performed to determine the ability of rebamipide to regulate lipid metabolism. As seen in Fig 3A, lipid droplets were generated by treatment with PMA and ox-LDL, however, co-treatment with rebamipide inhibited the formation of foam cells in a dose-dependent manner (Fig 3A), though rebamipide had little effect on cell viability (Fig 3B). Collectively, the results showed that rebamipide has the potential to control lipid metabolism.

### Rebamipide decreases levels of pro-inflammatory cytokines and controls the balance between Th17 and Treg cells

**Fig 4:**
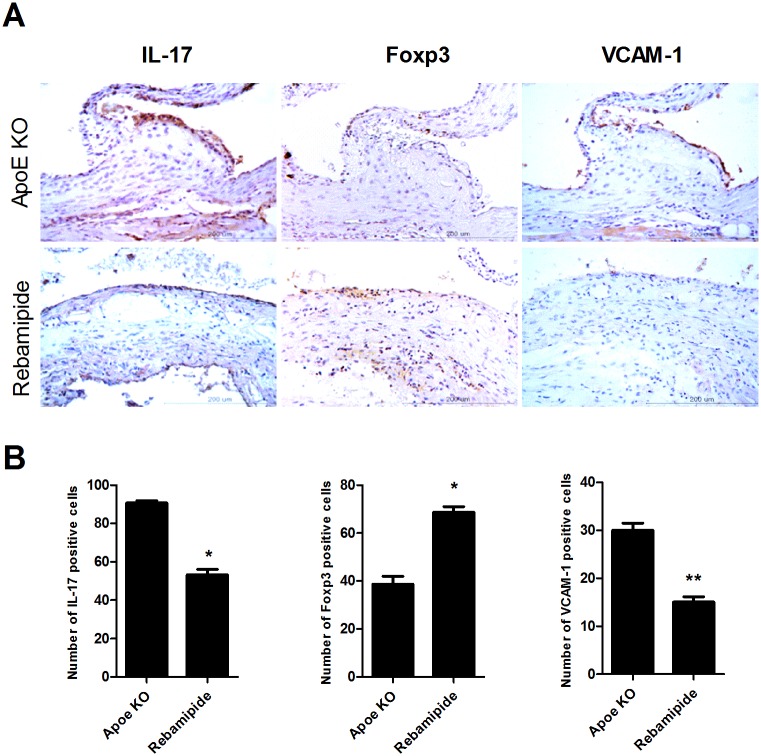
Regulation of IL-17 and Treg cells and suppression of cell adhesion. (A) The aorta isolated from ApoE-KO mice treated with or without rebamipide were subjected to immunochemical staining for IL-17, Foxp3 or VCAM-1. Scale bars: 200um. (B) IL-17, Foxp3 and VCAM-1 expression in atherosclerotic lesions from ApoE-KO mice treated with or without rebamipide. The number of cells was counted in four independent quadrants.

**Fig 5:**
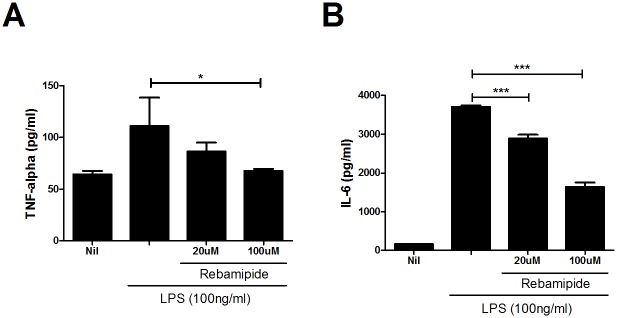
Rebamipide suppresses inflammatory cytokines. Normal B6 cells were isolated from the spleen, treated with LPS (100ng/ml) and incubated with or without rebamipide at the indicated dose for 3 days. Then, enzyme-linked immunosorbent assay (ELISA) was performed using the supernatants to measure the inflammatory cytokines TNF-α, IL-6.

Increased levels of AST or ALT are indicative of potential inflammation in vivo. Rebamipide showed a stabilizing effect on the levels of AST and ALT (Fig 2A), hence, we investigated the anti-inflammatory function of rebamipide *in vivo* by evaluating the change in inflammatory cytokines. Immunohistochemical data showed a significant decrease in IL-17 level in the rebamipide treated mice, as compared to ApoE-KO mice, while the level of Foxp3 was increased by rebamipide (Fig 4A). Graph also indicated that rebamipide increased the level of Foxp3 and suppressed the IL-17 and VCAM-1 expression (Fig 4B). Next, we confirmed the regulatory effect of rebamipide on pro-inflammatory cytokines *ex vivo*. Primary total splenocytes were stimulated with LPS for 72 hrs., and the cells were subsequently treated with 20μM or 100μM of rebamipide. The results of ELISA indicated that TNF-α and IL-6 were decreased by rebamipide treatment, as compared to the LPS treated control (Fig 5A–5C). IL-1β, however, did not show a dramatic change with rebamipide treatment.

**Fig 6:**
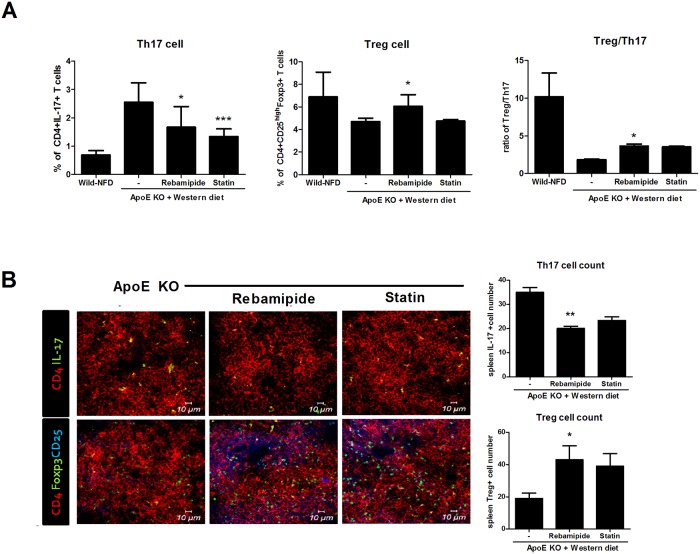
Rebamipide modified Th17/Treg balance. (A) Total splenocytes were isolated from rebamipide treated western-dieted ApoE-KO mice or control ApoE-KO mice. For FACS analysis, cells were stained with anti-CD4 and anti-IL-17 antibodies for Th17 cells, and with anti-CD4, CD25 and FoxP3 for Treg cells. (B) Spleen tissues from western-dieted ApoE-KO mice were also stained for Th17 or Treg cells and confocal microscope images were obtained. Bar indicates 10μm.

Our previous results showed that rebamipide decreased IL-17 and increased the expression of Foxp3 in the aorta of ApoE-KO mice on a western diet (Fig 4), which suggested that rebamipide does not only inhibit the inflammation but also upregulates the Treg cell population. To confirm the regulatory role of rebamipide on Th17/Treg balance, we investigated the change of the Th17 or Treg cell population by rebamipide *ex vivo*. Total splenocytes from ApoE-KO mice were treated with rebamipide or statin as the positive control. FACs showed that Th17 cells were increased in ApoE-KO mice fed a western diet but rebamipide and statin treatment decreased the Th17 cell population. Surprisingly, the decreased population of Treg cells in ApoE-KO mice was recovered by rebamipide treatment compared with western diet control mice. These change of Th17 or Treg cells led the increment of Treg/Th17 cell ration by rebamipide or statin (Fig 6A). Confocal staining of IL-17 or Foxp3 also showed that rebamipide and statin inhibited the secretion of IL-17 but upregulated Foxp3 expression (Fig 6B). Thus, these data confirmed the anti-inflammatory role of rebamipide as well as its ability to regulate the Th17/Treg balance in atherosclerosis in ApoE-KO mice.

### Rebamipide has potential ability to control inflammation-related disease

**Fig 7:**
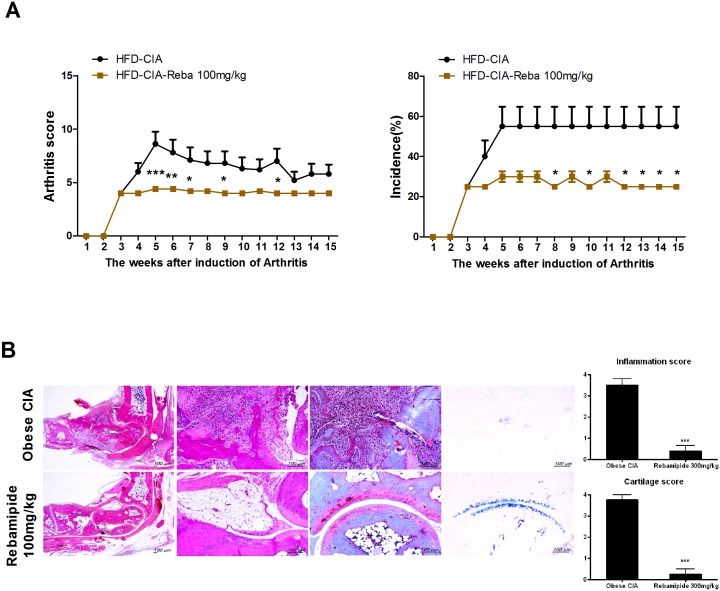
Rebamipide effect on obese arthritis in vivo. (A) B6 (7 weeks old) mice were divided into 2 groups (N = 5) and were fed a high fat diet and injected with type II collagen to induce obesity-rheumatoid arthritis (obese CIA). Mice were treated with rebamipide (100mg/kg) orally once a week for 6 weeks. The graphs show disease score and incidence of arthritis. (B) Joint tissues from obese CIA mice with or without rebamipide were stained with H&E, safranin O or Toluidine blue to determine the destruction of the joints. The graph of inflammation score and cartilage score show lymphocyte infiltration and joint destruction in obese CIA mice with or without rebamipide, respectively.

According to our data above, rebamipide is able to control inflammation in the hyperlipidemia condition in addition to improving lipid metabolism. Hyperlipidemia is accompanied by other inflammatory diseases such as fatty liver with inflammation or obesity-rheumatoid arthritis. To further investigate the effect of rebamipide, we determined the effects of rebamipide in the obese arthritis mouse model, which represents both lipid metabolic and inflammatory diseases. We established collagen-induced arthritis in mice with a high-fat diet (HFD-CIA), and mice were treated with or without rebamipide. As shown in Fig 7A, rebamipide dramatically decreased the arthritis score and incidence in the HFD-CIA model. Histochemical staining of the joint also confirmed that rebamipide decreased the destruction of the joint, as compared to the control mice (Fig 7B). Collectively, our data demonstrated that rebamipide has sufficient ability to relieve the hyperlipidemia condition followed by the balancing of Th17/Treg.

## Discussion

Rebamipide, a 2 (1H)-quinolinone analogue, is used for the treatment of gastroduodenal ulcer or gastritis. It induces the synthesis of prostaglandin, activates growth factors and ameliorates inflammation by oxidative stress [19]. Thus, rebamipide is a broad spectrum therapeutic agent for inflammatory diseases such as osteoarthritis, Sjogren syndrome, and Bechet’s disease [20–23]. Although rebamipide is also known to improve blood flow, remove free radicals and possess anti-inflammatory properties [24–26], its anti-inflammatory role in atherosclerosis accompanied by inflammation in hyperlipidemia condition has not been evaluated. We hypothesized that rebamipide improves lipid metabolism as well as inflammation and thus ameliorates atherosclerosis.

First, we confirmed that rebamipide decreased plaque formation in the aorta and improved hyperlipidemia in ApoE-KO mice. ApoE-KO mice on a western diet showed plaque formation within 8 weeks, however, the aorta from rebamipide or statin-treated mice showed improvements in plaque formation (Fig 1), which was confirmed through the recovered triglyceride (TAG) and LDL-cholesterol lipid metabolic bio-markers (Fig 2). Moreover, rebamipide’s ability to stabilize AST was superior to that of statin, a drug available for human atherosclerosis treatment. Rebamipide inhibited the formation of lipid droplets in foam cell assays using the human monocyte cell line, THP-1. Rebamipide effectively decreased oxidized ILD (Ox-LD)-induced lipid droplets (Fig 4). Previous studies demonstrated that rebamipide inhibits ox-LDL and MSU crystal-induced ROS and IL-1β secretion promoting foam cells [27, 28]. IL-1β is a key regulator of inflammation and the pathogenesis of vascular disease. As the stabilization of AST or ALT is indicative of anti-inflammation *in vivo*, we hypothesized that rebamipide controls inflammation in atherosclerosis.

We thus focused on the regulation of Th17 cells by rebamipide. Th17 has been highlighted as one of the inflammatory T cells in atherosclerosis [6, 7]. The level of IL-17 was increased in ApoE-KO mice on a western diet, and rebamapide dramatically decreased IL-17. Surprisingly, the administration of rebamipide upregulated the level of Foxp3, suggesting a regulatory role in the balance between Th17 and Treg cells in atherosclerosis (Fig 3A). Further study confirmed that the administration of rebamipide decreased Th17 cell population and improved Treg cell population *in vivo* (Fig 6A). We also observed the increase of Foxp3 level by rebamipide in the spleen of ApoE-KO mice (Fig 6B). Suppressed inflammatory cytokines such as TNF-α, IL-6, and IL-1β further confirmed our previous data (Fig 5).

In addition, rebamipide effectively decreased the VACAM-1 level (Fig 3a). VACM-1 is not expressed in normal conditions, and is a critical factor and marker in atherosclerosis [29, 30]. VCAM-1 provides robust adhesion and mediates the recruitment of rolling-type cells into atherosclerosis lesions depending on α_4_β_1_ integrin [31]. In ApoE-KO mice, the blocking of α_4_ integrin inhibits the recruitment of peritoneal macrophages in atherosclerosis [32]. Thus, down-regulation of VCAM-1 by rebamipide may contribute to anti-inflammation in ApoE-KO mice. Altogether, these data suggest that rebamipide is able to improve atherosclerosis and lipid metabolism, with immune-modulation and anti-inflammation functions.

The dual role of rebamipide in regulating lipid levels and inflammation could have potential therapeutic effects in obese-related inflammatory diseases. We tested the effects of rebamipide in obese arthritis using collagen-induced arthritis mice with obesity (obesity-CIA). Rebamipide significantly decreased the arthritis score and the incidence of arthritis in the obesity CIA model (Fig 7A). Immunohistochemical data indicated that joint destruction was also improved by rebamipide, as compared to the obesity CIA control (Fig 7B). This result in the obese arthritis model is indicative of the probability of a similar therapeutic effect by rebamipide in atherosclerosis; further *in vitro* or *in vivo* study is required to confirm the current findings.

## Conclusions

In this study, we showed that rebamipide reduces the development of atherosclerosis by controlling the balance between Th17 and Treg cells in western-dieted ApoE-KO mice. Rebamipide also suppressed the formation of lipid droplets *in vitro* and controlled lipid metabolism *in vivo*. Moreover, rebamipide decreased the severity of obese arthritis, another inflammatory disease that accompanies obesity. Thus, rebamipide could be a therapeutic agent to improve the progression of inflammation in metabolic diseases.
